# Use of Biomolecular Tools to Control the Labels of Ethnic Food Coming from Eastern Europe

**DOI:** 10.3390/foods13142181

**Published:** 2024-07-11

**Authors:** Alessandra Dalmasso, Daniele Pattono, Carla Bilewski, Federica Biolcati, Silvia Maida, Maria Teresa Bottero

**Affiliations:** 1 Dipartimento di Scienze Veterinarie, Università di Torino, Largo Braccini 2, 10095 Grugliasco, TO, Italy; daniele.pattono@unito.it (D.P.); f.biolcati@saccosrl.it (F.B.); s.maidavet@gmail.com (S.M.); mariateresa.bottero@unito.it (M.T.B.); 2 Independent Researcher, 10100 Turin, TO, Italy

**Keywords:** ethnic processed food, animal species identification, PCR, Sanger sequencing

## Abstract

In recent years, due to the large Romanian community present in Italy, the retail of foods coming from Eastern Europe has increased. The most common type of violation detected in these foods consists of incorrect labeling and species-replacement frauds. In this paper, the compliance of labels of 43 ethnic processed food coming from Eastern Europe and commercialized in Italy was evaluated by means of PCR and Sanger sequencing. Our data revealed 33% of non-compliant labels in samples containing swine, ruminants, and avian ingredients. These results demonstrate that PCR can be easily used for the identification of species in highly processed products, proving to be a rapid, effective, and economic method. On the other hand, samples reporting fish as ingredients highlighted the ineffectiveness of the applied sequencing protocol, due to the low informative property of targeted fragments or to the lack of consensus sequences in the case of uncommon species.

## 1. Introduction

Ethnic food can be defined as the food of a particular country that is culturally and socially accepted by consumers outside of the respective ethnic group (Kwon, 2015 [1]). The circulation in Europe of ethnic food is influenced by several factors, among which migration flows certainly play a crucial role (Fusco et al., 2015 [2]). In Italy, following Romania’s accession to the European Union in 2007, the Romanian community has become the largest among the foreign populations, with 1,081,836 citizens, accounting for 21% of the total foreign nationals legally residing in the country (5,141,341 individuals) (Italian National Institute of Statistics, 2024 [3]). Consequently, the retail of products from Eastern European countries has increased (D’Amico et al., 2014 [4]). Typically, these foods are prepared by companies in Eastern Europe and then sent to the Italian market, and labeled in Italian, as required by Regulation (EU) No. 1169/2011 [5] on food labeling.

Ensuring adherence to labeling regulations is a critical issue in the food supply chain due to its significant economic, health, and ethical implications. Concerns about economic fraud, such as the substitution of one ingredient for another similar but cheaper one, must be addressed, as well as potential health considerations arising from the absence of components that can affect individuals with specific sensitivities or allergies. Moreover, it is crucial to support consumers’ confidence in their dietary choices influenced by lifestyle factors or religious beliefs (Giusti et al., 2023 [6]). 

The most frequently reported violation of mandatory regulatory requirements consisted in (i) adulteration, referred to as the process of mixing or replacing an ingredient of high value with one of low value; (ii) mislabeling, which includes total or partial missing information or incorrect translation from the original languages and iii) document issues, which encompass falsified documents and traceability (European Commission’s Food Fraud Network, 2020 [7]). The 2022 annual report of the Alert and Cooperation Network (ACN) reported that “adulteration/product tampering” was the primary cause of food fraud notification (47.8%), followed by “misdescription/mislabeling/misbranding” (22.5%) and “document forgery” (9.3%) (ACN, 2022 [8]). 

To ensure label conformity in animal-origin foods, species identification through morphological examination may be useful when anatomical characteristics are preserved. Furthermore, a high proportion of ethnic foods has been usually processed and subjected to severe treatments during processing (i.e., canned foods) with consequent loss of ingredients integrity (Fujisaki et al., 2017 [9]; Xing et al., 2020 [10]). 

As a result, reliable diagnostic procedures are required to identify and/or authenticate these products (Dobrovolny et al., 2019 [11]). Several molecular techniques have been developed, initially focusing on protein fractions in foods. However, these methods have been largely discarded due to their low specificity and unsuitability for complex matrices subjected to processing (Raja Nhari et al., 2019 [12]). 

In contrast, DNA is more stable to thermal treatments, and it exhibits high inter-species and low intra-species variability, so it could be the suitable target to design species-specific tests (Bottero and Dalmasso, 2011 [13]). Moreover, because the DNA is relatively resistant under thermal treatments, the selection of small DNA fragments (<350 bp) as targets, improves the ability to analyze highly processed foods, where denaturing treatments can cause DNA fragmentation (Böhme et al., 2019 [14]). Additionally, the selection of a multicopy target molecule, such as mitochondrial DNA, enables the detection of a specific sequence even in a minimal amount (Bojolly et al., 2017 [15]). 

Several biomolecular methods including PCR (Thanakiatkrai et al., 2019 [16]), Sanger sequencing (Minoudi et al., 2020 [17]), PCR followed by digestion with restriction enzymes (PCR-RFLP) (Mata et al., 2020 [18]), real-time PCR (Kim and Kim, 2019 [19]) and melting curves analysis (Shi et al., 2020 [20]) have been used to authenticate highly processed food of animal origin. Among these techniques, PCR-RFLP exhibits a lack of protocol standardization, often requiring multiple enzymes to differentiate between several animal species (Bottero et al., 2011 [13]). Melting curve analysis through real-time PCR offers greater potential for automation since it requires no post-PCR manipulations, but it necessitates specific instruments and reagents, thereby increasing utilization costs. Conversely, the affordability of PCR equipment makes its application widespread in most laboratories, and the high accuracy of Sanger sequencing establishes it as the gold standard for all animal species identification analyses (Cichna-Markl and Mafra, 2023 [21]).

The aim of this study is to evaluate the label compliance of ethnic foods originating from Eastern European countries (Romania and Moldova). 

Firstly, a label inspection was conducted to confirm the presence and accuracy of labels in Italian; secondly, the declared animal species were evaluated using molecular methods based on PCR and Sanger sequencing.

## 2. Methods

### 2.1. Samples

A total of 42 ethnic canned foods from Eastern European countries (Romania and Moldova) were analyzed: in detail, 31 samples of processed meat foods, 3 of dairy products and 8 of canned fish products (Table 1). 

Samples were purchased from local retail markets located in Turin (Italy) and immediately transported to the laboratory, where they were stored at room temperature until DNA extraction. 

### 2.2. Label Inspection

A preliminary label inspection was conducted: the presence and the correctness of a label in the Italian language was verified. A native interpreter was asked to translate original language labels if the translation was missing.

### 2.3. DNA Extraction and Biomolecular Analyses

DNA extraction from all samples was performed using a DNeasy Blood & Tissue Kit (Qiagen, Hilden, Germany) with minor modifications customized for complex products, including an increase in the sample amount from 25 to 400 mg and a decrease in the final elution volume to 100 µL. 

DNA quantification was carried out using a NanoDrop-2000 (Thermo Fisher Scientific, Waltham, MA, USA).

All used primers and protocols are reported in Table 2.

Species-specific PCR-based protocols were applied for all samples, except for fish samples, whereby the species were identified by PCR followed by Sanger sequencing. To analyze highly denatured samples (canned products), primers allowing the amplification of reduced-length mitochondrial fragments were selected.

All processed meat samples were analyzed to detect specific DNA from swine, bovine, poultry, turkey, goat, and sheep species, as well as conserved DNA for the fish class. Additionally, two samples indicating the presence of duck meat on the label were specifically tested for this species. The three dairy products were tested exclusively using bovine, goat, and sheep specific protocols. Furthermore, eight samples containing fish were subjected to amplicon sequencing for species identification.

All PCR amplifications were performed in an ABI 2720 thermocycler (Applied Biosystems, Waltham, MA, USA) on a final volume of 50 µL containing: 20 mM Tris-HCl (pH 8.4); 1 unit of Platinum Taq DNA polymerase (Thermo Fisher Scientific); 0.2 mM each of dATP, dCTP, dGTP, and dTTP Stain (Thermo Fisher Scientific); 2 mM MgCl_2_; 25 pmol of each primer; and 50 to 250 ng of DNA template. After an initial denaturation step at 94 °C for 5 min, 35 cycles were programmed as follows: 94 °C for 1 min, annealing temperature for 1 min, 72 °C for 1 min. Annealing temperatures were selected following the authors’ instructions. A final extension at 72 °C for 5 min was set up.

Amplimers were resolved by electrophoresis on a 2.5% agarose gel stained with SYBR^TM^ safe DNA gel stain (Thermo Fisher Scientific).

The amplicons obtained with fish primers were purified by ExoSap treatment according to the manufacturer’s recommendations (Thermo Fisher Scientific). Forward and reverse sequencing reactions were performed using an ABI Prism BigDye Terminator Cycle Sequencing Ready Reaction Kit, version 1.1 (Thermo Fisher Scientific). The extended products were purified with DyeEx 2.0 Spin kit (Qiagen) and resolved by capillary electrophoresis using a SeqStudio™ Genetic Analyzer System (Thermo Fisher Scientific). The electropherograms were analyzed using Mega11 software (Tamura et al., 2021 [25]) and the sequences were submitted to BLAST similarity search software (BLAST+ 2.15.0) on the NCBI (National Center for Biotechnology Information) website (https://blast.ncbi.nlm.nih.gov/, accessed on 1 March 2024). 

## 3. Results and Discussion

In the present study, a survey on the conformity of labels of ethnic processed foods produced in Moldova and Romania and marketed in Italy was performed using PCR and Sanger sequencing.

Concerning the label inspection, it was observed that in 93% of the cases (39/42), an Italian label was present, and the translation was consistent with the original language, while the remaining samples presented only a label in Romanian. 

As regards the species-specific PCR, 14 out of 42 samples were found to be non-compliant (Table 3). Among these, undeclared additional species were detected in 10 samples of meat products. These results may be attributed to the production process, as food processing plants where different raw materials of animal origin are processed showed a certain susceptibility to cross-contamination, potentially due to inadequate cleaning systems. Consequently, the incomplete removal of residues from previous processing, combined with the high sensitivity of biomolecular methods capable of detecting even traces of DNA, increases the likelihood of detecting undeclared species.

Within our samples showing undisclosed additional species, cross-contamination was presumed in five meat samples (ID1, ID3, ID8, ID33, ID37) due to the similarity in value between the meats detected. In contrast, in the duck pâté sample (ID6), the presence of additional swine DNA is more likely the result of an intentional, even partial, substitution due to the low economic value of the added species. Similarly, Amaral et al. (2015) [26] found significant cases of adulteration involving the substitution of high-value game birds (such as partridge, pheasant, and quail) for lower-value poultry meat.

Furthermore, as already suggested by several authors, in the case of detecting undeclared DNA species, it is often not feasible to differentiate between an accidental contamination and an intentional addition (Guardone et al., 2017 [27]; Visciano et al., 2023 [28]). Indeed, DNA quantification could allow the distinction between a low-level contamination and an intentional addition of species in high quantities for economic reasons. However, quantifying DNA in food can be challenging, especially when dealing with mitochondrial DNA, considered the gold standard for animal species identification, as a variation exists in the number of mitochondria in different tissues (e.g., liver, muscle) (Bottero and Dalmasso, 2011 [13]). 

Interestingly, the presence of undeclared swine DNA in three samples (ID31, ID36, ID39) could be attributed to a cross-contamination or an intentional mislabeling aimed at bypassing the sanitary certification, which attests the absence of Classical Swine Fever (CSF) and African Swine Fever (AFS). In fact, due to the persistence of the CSF and ASF viruses in processed products, the commercialization of pork products from endemic countries (such as Eastern European countries) requires a specific certificate (Commission Implementing Regulation (EU) 2023/594 [29]). 

Furthermore, the presence of pork DNA could have serious implications for individuals adhering to dietary restrictions for health or religious beliefs, such as the prohibition of pork consumption for Jews and Muslims (Lubis et al., 2017 [30]).

Furthermore, one sample (ID13) showed the complete replacement of swine with bovine DNA. This could be explained by a labeling error or a reduced availability of pork meat due to the diffusion in Eastern Europe of CSF and ASF. In fact, to avoid the spread of CSF and ASF viruses, all pigs on the farms where the diseases were detected must be culled. Because of this measure, in Romania over 542,000 pigs affected by AFS were killed from July 2017 up to 2020 (Romanian National Veterinary Sanitary and Food Safety Authority, 2020 [31]). In 2022, Romania was the most affected EU country with 327 AFS outbreaks, representing 87% of the total EU outbreaks (European Food Safety Authority, 2023 [32]).

Finally, in samples containing “whey, milk powder or cream” as ingredients (ID5, ID10, ID27, ID39), the amplification of bovine DNA failed. As the DNA extraction and amplification from pure whey powder were successfully performed in previous research (Bottero et al., 2003b [33]), these results underline the complexity of DNA extraction when it is combined with additional ingredients. 

The challenges of the DNA extraction protocol are further compounded in samples reporting “egg yolk” on the labels (ID27, ID34), where the failure to detect poultry DNA could be attributed to both interference from other ingredients and to the localization of DNA within the germ cell. In fact, for eggs, some authors have proposed a specific DNA extraction procedure to improve the performance of biomolecular approaches (Nau et al., 2009 [34]). 

All three dairy products were found to be satisfactory, which was of major note because the DNA quantification in these samples would have been further complicated by the natural variability of somatic cells involved in DNA extraction.

All fish samples were successfully amplified with fish primers and subsequently subjected to Sanger sequencing (Table 4). Regarding the sequencing results, although high identity values (>98%) were obtained for all samples, unambiguous identification was achieved only in two samples containing *Salmo salar* (ID14 and ID27). Conversely, despite obtaining high identity scores (>99%) for four samples (ID 15, ID16, ID19, ID26), the identification was hindered by both the genetic relationship of species belonging to *Gadus* and *Scomber* genera, and the low informative properties of sequences due to their reduced length (Debenedetti et al., 2014 [35]). 

Finally, the reliability of the sequencing result was compromised when the species were genetically related, as in the case of two samples (ID17, ID18) labelled as “sprat”. The sequencing results showed a high similarity to sequences of *Sprattus sprattus* (98.81%), *Clupea pallasi* (98.21%) and *Clupea harengus* (98.21%), with the difference among the identity scores attributed to a single polymorphic nucleotide present in the amplified fragment (224 bp). With regard to this, Bottero and Dalmasso (2011) [13] suggested the amplification of longer fragments to increase the reliability of the sequencing results. However, when dealing with highly denatured food matrices, where DNA is fragmented into small fragments, it would be prudent to sequence other genes to strengthen our findings. 

## 4. Conclusions

The assurance of food label compliance with EU regulations is a crucial concern due to implications for consumer confidence and safety. This represents a significant challenge, particularly for mixed, processed, and heat-treated foods, such as canned ethnic foods which are usually processed to extend their shelf life and facilitate their distribution worldwide.

In this study, ethnic foods coming from Eastern European countries were studied: the results showed a low rate (7%) of formal deficiencies concerning label translations; furthermore, there was a higher rate of non-compliance (33%) in animal origin ingredients detected by biomolecular methods.

These results demonstrate the effectiveness of DNA-based methods in identifying species in highly processed foods where ingredient integrity has been compromised, overcoming the limitations of other methods as protein-based approaches.

However, they also highlight challenging issues that require attention, such as the high sensitivity of biomolecular analyses, which makes it difficult to distinguish between cross-contamination and fraudulent activities, especially when DNA quantification is hindered by the type of molecular target (e.g., mitochondrial DNA) and in certain matrices (e.g., milk).

Lastly, despite the abundance of sequences available in databases obtained by modern massive sequencing techniques, even the identification of genetically related species is not always possible due to the similarity of mitochondrial genomes.

## Figures and Tables

**Table 1:** Samples.

ID	Sample	Ingredients of Animal Origin Reported on Label
1	Chicken pâté	Chicken liver, chicken meat
2	Chicken meat	Chicken meat
3	Chicken pâté	Chicken meat, chicken liver, chicken skin
4	Turkey pâté	Turkey skin, turkey liver, turkey and chicken mechanically separated meat
5	Duck pâté	Chicken mechanically separated meat, chicken liver, milk serum, egg albumen, duck meat
6	Duck pâté	Duck liver and meat
7	Canned meat	Pork, beef, and turkey meat
8	Pork pâté	Pork liver, bacon, rind
9	Pork pâté	Pork liver, lard, rind
10	Pork liver pâté	Pork liver, bacon, milk powder
11	Pork pâté	Pork liver, bacon
12	Pork with sauerkraut	Pork meat
13	Stuffed cabbage	Pork meat
14	Salmon fillets	Salmon
15	Cod liver	Cod liver
16	Cod liver pâté	Cod liver, cod eggs
17	Sprats in oil	Sprats
18	Sprats with tomato sauce	Sprats
19	Mackerel with rapeseed oil	Mackerel
20	Pork and chicken sausage	Turkey, pork and chicken meats
21	Pork and beef sausage	Pork and beef meat, bacon, rind, pork liver and heart
22	Stuffed pork	Pork meat, bacon
23	Boneless beef	Beef meat, pork rind
24	Pork pâté with lard	Pork liver
25	Chicken pâté with lard	Chicken and pork meat
26	Cod liver pâté	Cod liver
27	Salmon	Salmon, egg yolk, skimmed milk
28	Cheese with ham	Pork ham, milk, butter
29	Fresh cheese	Bovine milk
30	Pork liver pâté	Pork liver, bacon, rind
31	Chicken pâté	Chicken liver, chicken meat
32	Beans with pork shank	Pork shank
33	Sausages with beans	Pork meat, lard
34	Tripe	Beef tripe, crem, egg yolk
35	Pork liver pâté	Pork liver and lard
36	Beef meat	Beef and poultry meats
37	Pork meat	Pork meat, lard, rind and mechanically separated meat
38	Sheep’s cheese	Sheep’s milk
39	Chicken pâté	Chicken liver and meat, milk powder, cream
40	Cheese with ham	Bovine milk, butter, pork
41	Goat’s cheese	Goat’s milk
42	Sheep’s cheese	Sheep’s milk

**Table 2:** Primers used for PCR protocols.

Identified Species	Primers	Gene Targets	Amplicon Lenght	References
Swine (*Sus scrofa*)	Sense: 5′ CTACATAAGAATATCCACCACA 3′ Antisense: 5′ ACATTGTGGGATCTTCTAGGT 3′	12s rRNA, tRNA val	290 bp	Dalmasso et al. (2004) [22]
Bovine (*Bos taurus*)	Sense: 5′ GTACTACTAGCAACAGCTTA 3′ Antisense: 5′GCTTGATTCTCTTGGTGTAGAG 3′	12s rRNA, 16s rRNA	256 bp	Bottero et al. (2003) [23]
Goat (*Capra hircus*)	Sense: 5′ CGCCCTCCAAATCAATAAG 3′ Antisense: 5′AGTGTATCAGCTGCAGTAGGGTT 3′	12s rRNA, 16s rRNA	326 bp	Bottero et al. (2003) [23]
Sheep (*Ovis aries)*	Sense: 5′ATATCAACCACACGAGAGGAGAC 3′ Antisense: 5′TAAACTGGAGAGTGGGAGAT 3′	12s rRNA, 16s rRNA	172 bp	Bottero et al. (2003) [23]
Chicken (*Gallus gallus)*	Sense: 5′ACATAGAACAAACGAAAAAGGATGTG 3′ Antisense: 5′CGTCTTAAAGTGAGCTTAGGGCG 3′	12s rRNA	95 bp	Martín et al. (2007) [24]
Turkey (*Meleagris gallopavo*)	Sense: 5′ CCACCTAGAGGAGCCTGTTCTRTAAT 3′ Antisense: 5′ TTGAGCTCACTATTGATCTTTCAGTTT3′	12s rRNA	122 bp	Martín et al. (2007) [24]
Duck (*Anas plathyrincos*)	Sense: 5′ CATAATTAATACCCTGTAAATGCC 3′ Antisense: 5′ TCCAGTATGCTTACCTTGTTACGAC 3′	12s rRNA	64 bp	Martín et al. (2007) [24]
Fish	Sense: 5′ TAAGAGGGCCGGTAAAACTC 3′ Antisense: 5′ GTGGGGTATCTAATCCCAG 3′	12s rRNA	224 bp	Dalmasso et al. (2004) [22]

**Table 3:** Results of PCR for non-compliant samples.

ID	Ingredients of Animal Origin Reported on Label	Swine	Bovine	Poultry	Turkey	Duck	Fish
1	Chicken liver, chicken meat	−	−	+	+	−	−
3	Chicken meat, chicken liver, chicken skin	−	−	+	+	−	−
5	Chicken, mechanically separated meat, chicken liver, milk serum, egg albumen, duck meat	−	−	+	−	+	−
6	Duck liver and meat	+	−	−	−	+	−
8	Pork liver, bacon, rind	+	−	−	+	−	−
10	Pork liver, bacon, milk powder	+	-	−	−	−	−
13	Pork meat	−	+	−	−	−	−
27	Salmon, egg yolk, skimmed milk	−	−	−	−	−	+
31	Chicken liver, chicken meat	+	−	+	−	−	−
33	Pork meat, lard	+	+	-	−	−	−
34	Beef tripe, cream, egg yolk	−	+	-	−	−	−
36	Beef and poultry meats	+	+	+	−	−	−
37	Pork meat, lard, rind, and mechanically separated meat	+	−	+	−	−	−
39	Chicken liver and meat, milk powder, cream	+	−	+	−	−	−

The boxes highlighted in grey color report non-compliance results.

**Table 4:** Results of PCR for fish, followed by Sanger sequencing of amplicons.

ID	Ingredients of Animal Origin Reported on Label	Preliminary PCR for Fish	Sequencing results		
			Query Coverage	Identities	Identified Species
14	Salmon *(Salmo salar)*	**+**	100%	99.39%	*Salmo Salar*
15	Cod liver (*Gadus morhua*)	**+**	100% 100%	99.39% 99.39%	*Gadus morhua* *Gadus macrocephalus*
16	Cod liver, cod eggs (*Gadus morhua*)	**+**	100% 100%	99.39% 99.39%	*Gadus morhua* *Gadus macrocephalus*
17	Sprats (*Sprattus sprattus)*	**+**	100% 100% 100%	98.81% 98.21% 98.21%	*Sprattus sprattus* *Clupea pallasii* *Clupea harengus*
18	Sprats (*Sprattus sprattus*)	**+**	100% 100% 100%	98.81% 98.21% 98.21%	*Sprattus sprattus* *Clupea pallasii* *Clupea harengus*
19	Mackerel (*Sgomber japonicus*)	**+**	100% 100%	100% 100%	*Scomber japonicus* *Scomber australasicus*
26	Cod liver (*Gadus morhua*)	**+**	100% 100%	99.39% 99.39%	*Gadus morhua* *Gadus macrocephalus*
27	Salmon (*Salmo salar*), egg yolk, skimmed milk	**+**	100%	99.39%	*Salmo salar*

## Data Availability

The original contributions presented in the study are included in the article, further inquiries can be directed to the corresponding author.
